# Polyimide-On-Silicon 2D Piezoelectric Micromachined Ultrasound Transducer (PMUT) Array

**DOI:** 10.3390/s23104826

**Published:** 2023-05-17

**Authors:** Sanjog Vilas Joshi, Sina Sadeghpour, Michael Kraft

**Affiliations:** 1 Department of Electrical Engineering (ESAT-MNS), KU Leuven, 3000 Leuven, Belgium; sanjogvilas.joshi@kuleuven.be (S.V.J.);; 2 Leuven Institute for Micro- and Nanoscale Integration (LIMNI), KU Leuven, 3000 Leuven, Belgium

**Keywords:** piezoelectric thin films, PZT, piezo-mems, ultrasound transducers, PMUT, medical imaging

## Abstract

This paper presents a fully addressable 8 × 8 two-dimensional (2D) rigid piezoelectric micromachined ultrasonic transducer (PMUT) array. The PMUTs were fabricated on a standard silicon wafer, resulting in a low-cost solution for ultrasound imaging. A polyimide layer is used as the passive layer in the PMUT membranes on top of the active piezoelectric layer. The PMUT membranes are realized by backside deep reactive ion etching (DRIE) with an oxide etch stop. The polyimide passive layer enables high resonance frequencies that can be easily tuned by controlling the thickness of the polyimide. The fabricated PMUT with 6 µm polyimide thickness showed a 3.2 MHz in-air frequency with a 3 nm/V sensitivity. The PMUT has shown an effective coupling coefficient of 14% as calculated from the impedance analysis. An approximately 1% interelement crosstalk between the PMUT elements in one array is observed, which is at least a five-fold reduction compared to the state of the art. A pressure response of 40 Pa/V at 5 mm was measured underwater using a hydrophone while exciting a single PMUT element. A single-pulse response captured using the hydrophone suggested a 70% −6 dB fractional bandwidth for the 1.7 MHz center frequency. The demonstrated results have the potential to enable imaging and sensing applications in shallow-depth regions, subject to some optimization.

## 1. Introduction

Ultrasound transducers have traditionally utilized bulk piezoelectric actuation, which is a well-established technology in the medical imaging market [1]. However, this approach has several drawbacks. For instance, fabrication techniques such as mechanical dicing can limit the interelement pitch, thus restricting performance [2]. In addition, a bulky design requires high operating voltages, and the resonance frequency is solely dependent on device thickness, leaving little room for design variations [3]. Moreover, a thick piezoelectric layer results in poor acoustic matching and requires multiple matching as well as backing layers [1].

The trend towards portable and point-of-care devices has increased the demand for miniaturized and integrated electronic components, including more energy-efficient ultrasound transducers for imaging applications. Micromachined ultrasound transducers (MUTs) have emerged as a promising solution, offering a small form factor, high compatibility for mass production, and reduced power consumption. In particular, capacitive micromachined ultrasonic transducers (CMUTs) have demonstrated improved coupling coefficients and bandwidths, making them a milestone in the field of ultrasonic imaging since their introduction in 2000 [4,5,6]. CMUTs operate by creating vibrations through electrostatic forces between electrodes separated by a dielectric membrane and a vacuum cavity, which actuates the device in flexural mode. However, CMUTs have the drawback of requiring high operating voltages.

Piezoelectric thin-film actuation has gained popularity due to its low actuation voltage and higher actuation force [7]. This makes it a viable option for various MEMS devices and applications, such as inkjet printer heads [8], MEMS scanning mirrors [9], ultrasonic motors [10], RF resonators [11], and acoustic generators [12]. Recently, the use of piezo-electric thin films in piezoelectric micromachined ultrasonic transducers (PMUTs) has demonstrated satisfactory acoustic pressures for ultrasound-based imaging at relatively low voltages. A PMUT element consists of a membrane with a piezoelectric thin film. It is driven by applying an excitation voltage between the top and bottom electrodes of the piezoelectric layer. The applied electric field generates in-plane stress in the active piezoelectric layer. This causes out-of-plane membrane displacements, producing a pressure wave in the outer medium. Unlike electrostatic technology, PMUTs do not require a DC biasing voltage. Moreover, their membrane geometry can be tailored to achieve the required output acoustic pressures at desired frequencies [13].

A downside of PMUTs is their low electro-mechanical coupling coefficients, which are limited due to piezoelectric thin-film properties. This can result in lower bandwidth and hinder their applicability in applications requiring high bandwidth, such as medical imaging. We have previously shown that using polyimide in the PMUT membrane improves the bandwidth and tends to widen it [14,15]. Likewise, this paper uses polyimide to introduce damping and improve the bandwidth. Another downside of PMUTs stems from the typical materials used in their fabrication, such as AlN [16] and PVDF [17]. These have a weaker e_31_ piezoelectric response compared to PZT, resulting in low transmit sensitivity even at the resonance frequency. To overcome this challenge, our fabrication method employs a PZT thin film that boasts excellent piezoelectric properties [18].

Our method benefits from an in-house PZT solution-making process and deposition procedures to reduce costs. For further cost reduction, we used standard silicon wafers instead of more expensive SOI wafers. SOI technology is a mature and convenient PMUT fabrication technique because of the silicon device layer with a desired thickness and buried oxide etch stop during backside etching, which makes it easy to realize the membranes [13]. Furthermore, our fabrication process based on polyimide membranes is much simpler compared to the reported processes on conventional silicon wafers. Our process manages to keep the piezo layer away from the neutral axis, providing necessary bending moments for actuation and sensing [19]. Previously reported PMUT fabrication processes on conventional silicon wafers include back-side sloped sidewall cavity etching [20], front-side isotropic cavity etching with a via hole [21], sacrificial layer etching [22], etc. These processes lack a passive layer in the membrane, which is required to keep the piezo layer away from the neutral axis. A recent method based on hydrogen annealing [23] has a passive layer in the membrane, but it is a relatively more complicated fabrication process.

In our work, we fabricated and characterized a 2D PMUT array on a silicon wafer with a polyimide membrane without compromising on resonance frequency. The work is presented in the paper with the following organization. The next sections discuss the device design and finite element simulations carried out in COMSOL. In the following, the methodologies of PZT processing and PMUT fabrication are briefly described. Then, the material and electrical characterizations of PZT thin film as well as PMUT are explained. Air and water are the acoustically closest environments where PMUTs operate. Hence, the operation of our PMUTs is evaluated in-air as well as underwater, which can help optimize the design and performance of the transducer for specific applications in the future. The evaluations include LDV, and hydrophone measurements supported by finite element simulations, theoretical calculations, and reported data. Note that there are two crucial factors regarding transducer arrays that are not addressed much in the literature and are touched upon in this paper: (i) interconnected design to address each individual element (full addressing) in 2D arrays [19]; and (ii) inter-element crosstalk.

## 2. Device Design

**Figure 1:**
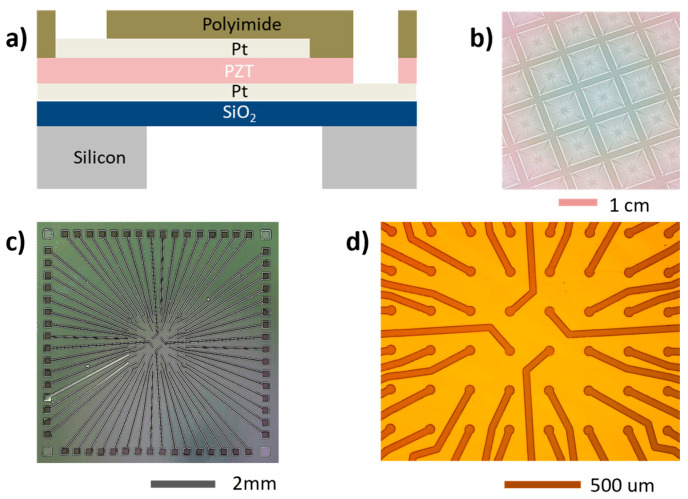
(**a**) Schematic of a PMUT element (not to scale). (**b**) Two-dimensional PMUT array on the wafer. (**c**) Design of the demonstrated 8 × 8 array. (**d**) Zoomed-in view.

Our proposed single PMUT element consists of a polyimide passive layer in the membrane situated above the top electrode and the active piezoelectric (PZT) layer, as shown in Figure 1a. Note that the polyimide passive layer arrangement in the membrane is different from the usual silicon passive layers in SOI PMUT technology. In an SOI PMUT, the passive silicon device layer of the SOI wafer is below the active piezoelectric layers. Another thing to note is that an SOI PMUT’s silicon passive layer keeps the neutral axis outside the PZT (the neutral axis should be outside the piezo layer to ensure a good sensitivity [19]), and here, polyimide does the same. By using polyimide, vibrations generated by the PZT layer are damped, which helps in widening the bandwidth. Additionally, the mass of the medium can effectively dampen the membrane vibration (thus improving fractional bandwidth) when the lateral dimension of the membrane is smaller than the wavelength; thus, 100 µm was chosen as the diameter of the PMUT membrane [14].

The underwater resonance frequency was designed to be 2–2.5 MHz so that the devices can be used for applications such as biomedical imaging. Studying PMUTs in water can provide important insights into their behavior in such applications as water has similar acoustic properties as the tissue medium [14]. In the array design, a half-wavelength pitch of 300 µm was chosen to avoid imaging artifacts and the creation of side lobes in the beampattern [3]. A fully addressed 8 × 8 array was designed, resulting in an around 2.4 mm × 2.4 mm aperture size. Fully addressable means that there is a dedicated top electrode pad for exciting each individual PMUT element. This presents a design challenge, especially for 2D array interconnects, as a transducer with m rows and n columns requires m × n interconnect lines for operation [19]. The proposed novel and scalable design of the top electrode interconnects is shown in Figure 1b–d. Including the interconnects and bond pads, the chip size is 1 cm × 1 cm. This design can be scaled to a greater number of elements by reducing the line width and drawing more lines between two adjacent top electrodes. A summary of the crucial design parameters (such as lateral dimension, pitch, etc.) for the PMUT is given in Table 1.

An advantage presented by this technology is the resonance frequency tunability by means of changing the polyimide thickness. The resonance frequency of the PMUT in the air is given by Equation (1) below. Here, *D* is the flexural rigidity of the membrane and *a* is the radius of the membrane. ρ*_i_* and *t_i_* are the densities and thicknesses of the materials used in the membrane stack. *k* is the proportionality constant depending on aspects of the PMUT design such as clamping conditions, active layer arrangement, etc. and was chosen per 10.4, based on [24]. Flexural rigidity, *D*, is the cubic function that can be shown as an integral (in Equation (2)) where *z*_0_ is the neutral axis position calculated using Equation (3). *Y_z_* is Young’s modulus of the material present at the *z* position. The linear mass density in the denominator of the first equation and the linear function of thickness. Basically, increasing the thickness makes the membrane effectively stiffer and increases the resonance frequency. Equation (4) shows that the mass of the water medium reduces the resonance frequency in comparison with the air medium. However, the dependence on the layer thickness, *t_i_*, is normally weak (also applies to our case) and follows a *t*^0.5^ relationship when the lateral dimensions are significantly larger (the second term is much larger than 1 in this case.). In essence, the combined effect of both equations is the increase in the underwater resonance frequency with the increase in the thickness of the polyimide layer.

We also theoretically calculated the PMUT resonance frequency underwater using these equations. The values of the used parameters are in Table 2. When 6 µm thick polyimide is used, the position of the neutral axis is found around 2 µm in height from the bottom of the membrane and is outside the PZT layer. Next, the flexural rigidity was calculated to be 740 nN.m, whereas the linear mass density was calculated as 17.12 g/m^2^. Using Equation (1), the in-air resonance frequency was estimated to be 4.35 MHz. Equation (4) was further used to find the underwater resonance at 2.35 MHz. Since 6 µm polyimide resulted in a resonance frequency between 2 and 2.5 MHz, we continued with this thickness for fabrication. Calculating using a 3 µm polyimide thickness resulted in 2.3 MHz and 1.1 MHz resonance values in air and underwater, respectively, validating our claims regarding resonance frequency tunability.

(1) $$f_{r}=\frac{1}{2\pi }\frac{k}{a^{2}}\sqrt{\frac{D}{\sum_{i}^{}\rho_{i}\cdot t_{i}}}\propto \frac{t}{a^{2}}$$

(2) $$D=\int_{0}^{h}\frac{Y_{z}(z-z_{0})2}{1-v_{z}^{2}}dz$$

(3) $$\int_{0}^{h}Y_{z}(z-z_{0})dz=0$$

(4) $$\frac{f_{r,water}}{f_{r,air}}=\frac{1}{\sqrt{1+0.84\frac{\rho_{water}.a}{\sum_{i}^{}\rho_{i}\cdot t_{i}}}}$$

## 3. COMSOL Simulations

The acoustic pressure simulations for the single PMUT element (not for the array) were carried out in a water medium with the following goals: (1) to validate the impact of the polyimide membrane passive layer on the transducer resonance frequency, which is the central design idea in this paper; (2) to provide more details on transducer operation such as the mode of vibration and the positioning of the neutral axis; and (3) to show the evolution of axial and lateral acoustic pressure profiles along with membrane vibration. Precise measurement of such profiles requires setups with position control and, moreover, such measurements can present difficulties due to noise where simulation helps provide more details. The simulations also support the LDV and hydrophone measurements.

**Figure 2:**
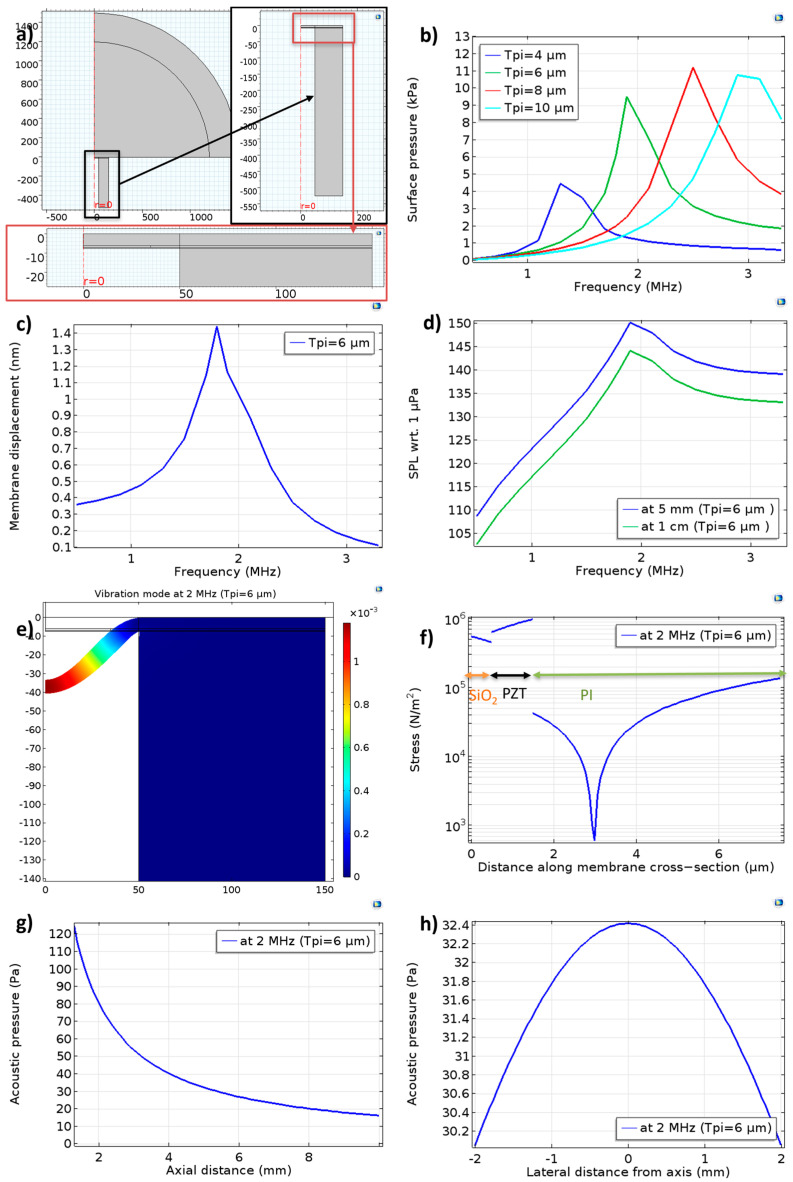
COMSOL simulation of a single PMUT element underwater: (**a**) Simulation geometry with zoomed−in views. The PMUT axis is indicated by r = 0, where r is the radial coordinate. (**b**) Simulation of surface pressure response with polyimide thickness as a parameter. For the PMUT element with 6 µm polyimide, (**c**) membrane displacement and (**d**) on−axis sound pressure level (SPL). Additionally, at 2 MHz, (**e**) vibration mode and (**f**) stress in the membrane along the axis of the PMUT, as well as (**g**) pressure decay with axial distance and (**h**) lateral pressure distribution at 5 mm.

A COMSOL 2D axisymmetric model was used to carry out these simulations. PMUT is an axisymmetric device, hence axisymmetric simulations can be used to drastically reduce the simulation time. Figure 2a shows the rectangular geometry. If we view the rectangular geometry in 3D, it is the same circular membrane. The materials, anchors, and actuation boundaries are also defined in a way that reflects the actual fabricated device. Based on COMSOL documentation, axisymmetric rectangle geometry has no impact on our studies involving first-mode resonance, which is a completely axisymmetric mode. Non-symmetric modes and non-linearities with respect to the axis are indeed difficult to simulate with such a representation.

The parameters from Table 1 and Table 2 were used in the simulation model. For the simulations, the electrodes were not separately defined. The PZT was actuated with a 50 mV electric potential difference between the top and bottom boundaries with the electrostatics module of COMSOL. This setting closely matched the performance of the fabricated PMUT when it was actuated with 1 V. The discrepancy in voltage is due to the difference in the piezoelectric and elastic properties of the PZT in the fabricated and simulated devices. The piezo material chosen in the COMSOL models, PZT-5H, comes with defined properties including piezoelectric and elastic coefficient matrices. However, we experimentally prepared a PZT thin film, the properties of which (such as the e31 coefficient) are not yet characterized. The properties of a thin film can be quite different from those of their bulk counterpart and are on the lower side. Hence, to achieve behavior at resonance similar to that in experimentation, 20-fold lower voltages were used in the simulation model compared to the ones used in the actual experiments.

The mesh and the perfectly matching layer (PML) were designed according to the COMSOL documentation [25]. The mesh size in the water domain ensured that the smallest wavelength was resolved by at least 6 elements, whereas the boundary layer thickness was chosen to be 20 times lower. The resolution in the narrow region (for instance, the piezo layer) was set to 2. For convergence testing, we tried parameterizing the maximum mesh size in the solid mechanics domain. However, because of the narrow region resolution condition, all meshes resulted in the same result, as this did not change the meshing significantly.

As a first step, a parametric frequency sweep was performed, and the underwater surface pressures of the PMUTs with various polyimide thicknesses are determined (Figure 2b). Moreover, we can identify the resonance frequency from the peak of surface pressure. This shows that 6 µm of polyimide in the passive layer results in an around 2 MHz resonance frequency underwater, hence confirming our choice of polyimide thickness for fabrication (except for the demonstration of the frequency tunability achieved using this design approach, where a 3 µm thickness was used). The simulation result again confirms that the resonance frequency increases if we increase polyimide thickness.

For the PMUT with 6 µm of polyimide, membrane displacement and on-axis transmitted pressures was determined (Figure 2c,d). These also confirmed a resonance frequency of around 2 MHz. Further results were obtained with 6 µm of polyimide at 2 MHz. Figure 2e shows the symmetric vibration mode. In Figure 2f, the stress in the membrane along the axis of the PMUT for this vibration mode is shown. The neutral axis (zero stress) can be seen close to but outside of the PZT film. The maximum stress is found at the anchors and in the PZT film as expected (not shown here). Compared with the SOI technology, the neutral axis in this design would be closer to piezoelectric owing to the low modulus of polyimide compared with silicon. This polyimide-on-PZT design would result in a smaller bending moment, negatively affecting the sensitivity [19]. This can be partially dealt with by improving the e_31_ coefficient with piezoelectric thin film process optimization and electronic focusing with a higher number of array elements [3]. Figure 2g shows pressure responses at the PMUT resonance as a function of axial distance whereas Figure 2h shows the pressures in the plane at 5 mm from the PMUT element. They indicate that the PMUT can produce about 32 Pa/V at 5 mm on the axis. Moreover, the pressure does not drop significantly within a 2 mm lateral distance. This implies that an electronically focused array of 64 elements can transmit around 64 times the pressure of a single element, amounting to around 2 kPa/V pressure at 5 mm.

## 4. Device Fabrication

**Figure 3:**
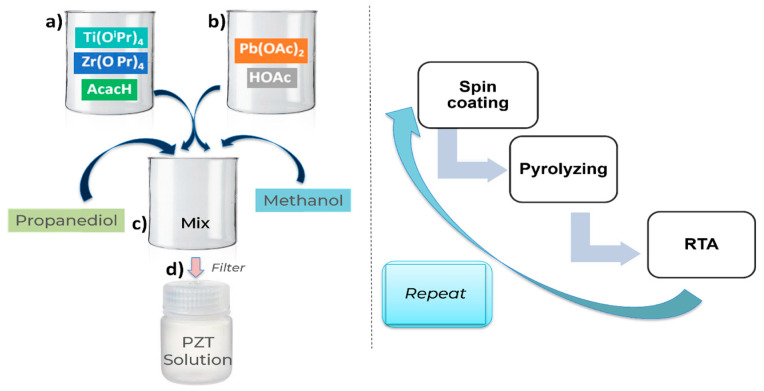
Left: recipe of making PZT solution—(**a**) In a beaker, Zr and Ti precursors are refluxed with acetylacetone in stochiometric amounts. (**b**) In another beaker, 10% of excess Pb precursor is refluxed with acetic acid. (**c**) Mixing the two solutions is followed by the addition of a sol stabilizer and solvent. (**d**) Mixing and filtration of the solution in a stock bottle. Right: PZT thin film deposition procedure: spin coating, pyrolysis, and RTA are repeated 4 times in a cycle.

Figure 3 shows the sol–gel deposition method for PZT thin film. As a first step, a PZT stock solution was prepared. Lead acetate, zirconium propoxide (70 wt.% solution in 1–propanol), and titanium isopropoxide were used as precursors. Acetic acid, acetylacetone, and propylene glycol were used as stabilizers and additives. Lastly, methanol was used as a solvent. The sequence of steps is described in Figure 3 (left). PZT was deposited on the Pt bottom electrode via repeated spin coating, pyrolysis, and rapid thermal annealing (RTA), with the parameters described at the right of Figure 3. Four iterations were performed to obtain a 1 µm film.

**Figure 4:**
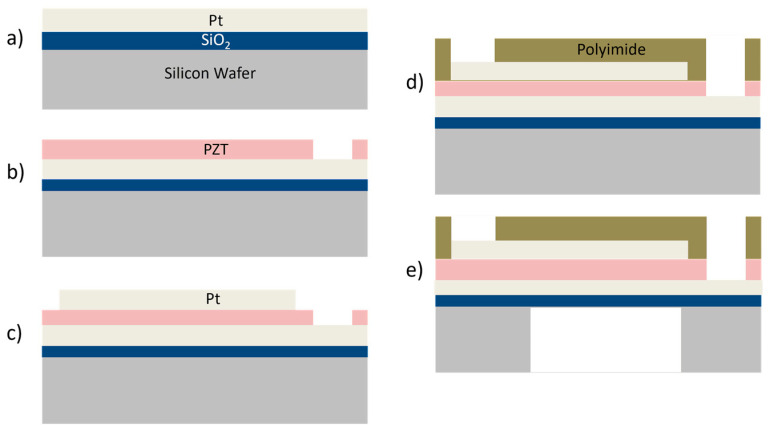
PMUT device fabrication process—(**a**) Oxide growth followed by deposition of the Pt as the bottom electrode. (**b**) Deposition and patterning of the PZT layer. (**c**) Patterning the top electrode. (**d**) Deposition and patterning of the polyimide passive layer. (**e**) Realizing the membrane via DRIE.

The 100–silicon wafer was prepared with 500 nm of oxide via wet oxidization in an oxidation oven. This was followed by sputtering for the 200 nm Pt bottom electrode. The oxide serves as an etch stop in the final DRIE process, whereas (111) oriented Pt serves as a seed layer for the PZT deposition process. Using the sol–gel method, 1 µm PZT was deposited on top of the Pt bottom electrode. The PZT was patterned using a previously reported wet etching procedure to access the bottom electrode [26]. Then, the top Pt layer was patterned using the liftoff for the electrodes covering a width of about 70% of the membrane diameter and for the interconnects. Once the active layers were prepared, polyimide (PI2611, HD Microsystems) was spun to serve as the membrane material and was patterned to access both electrode pads. Finally, the wafer backside was patterned using DRIE to realize the membranes and complete the fabrication. The fabrication process flow is shown in Figure 4, whereas the summary of materials processing involved in the fabrication is presented in Table 2.

## 5. Characterization Results and Discussion

### 5.1. Material and Electrical Characterization

**Figure 5:**
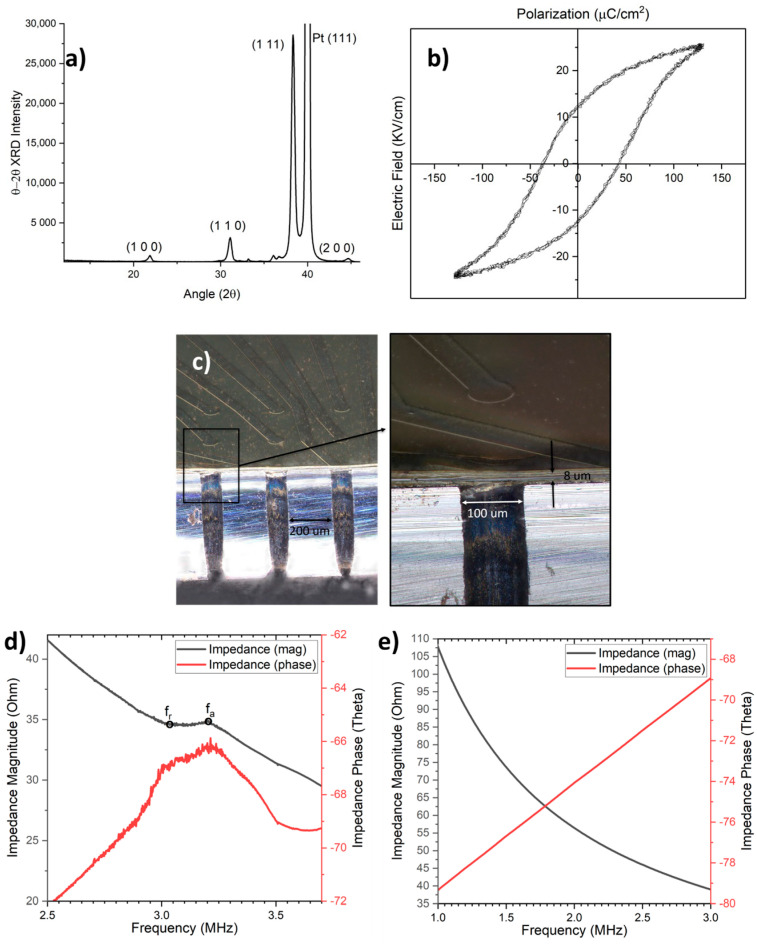
(**a**) XRD of the PZT. (**b**) P−E hysteresis loop of the PZT. (**c**) High−resolution optical microscope image of the high-frequency PMUT array membranes with 6 µm polyimide and 1 µm PZT. Zoomed−in view to the right. (**d**) The impedance of the single PMUT showing resonance (f_r_) and antiresonance frequencies (f_a_). (**e**) The impedance of the single PMUT underwater.

During fabrication, just after the PZT deposition step, the X-ray diffraction (XRD) pattern of the PZT thin film was obtained, showing (111)–orientated PZT (peak at 38°) without any pyrochlore phase (normally around 29°) (Figure 5a). The sharp peak around 40° is due to platinum (111) and was cut out, as it would otherwise diminish the important PZT peaks. Further characterization of the PZT involved P–E hysteresis loop measurements (Figure 5b). This was carried out after top electrode deposition. For this measurement, a Sawyer–Tower circuit was assembled where the PZT sample and a capacitor (C_0_) of a known value were connected in series. The value of the sample capacitor (~100 times the PZT capacitance) was chosen such that almost 99% of the applied voltage dropped across the PZT capacitor. From the applied voltage, the electric field was determined by considering the PZT thickness. Polarization charge density across the PZT capacitor was determined by dividing the charge across the capacitor of known value, C_0_V_0_, (same charge as the PZT capacitor in series) by the area of the PZT capacitor. A remnant polarization and coercive field of 12 µc/cm^2^ and 40 kV/cm, respectively, compared well with recent reports [27]. Here, note that our PZT thin film can stand up to 40 Vpp without breakdown, which defines the limit on the applied driving voltages. However, during the P–E loop measurements, a voltage of up to 30 Vpp was used to be on the safe side.

After the electrical characterization of the PZT, the device fabrication was completed. Figure 5c shows a high-resolution optical microscope image of the fabricated PMUT. The array membranes with 6 µm of polyimide and 1 µm of PZT show almost straight sidewalls. This demonstrates good fabrication quality. Next, while analyzing the device electrically, the capacitance of a single PMUT element was measured to be around 2.5 nF. However, note that this also includes the significant contribution of parasitic capacitance due to interconnects, which affects the performance of the PMUT and will be dealt with in future designs. Furthermore, the impedance of the PMUT device was measured with an impedance analyzer (6500 B, Wayne Kerr Electronics). The in–air impedance data shown in Figure 5d suggest a ~14% effective coupling coefficient. The effective coupling coefficient can be calculated from the resonance (f_r,_ taken as 3 MHz) and antiresonance frequencies (f_a,_ taken as 3.2 MHz) as (f_a_^2^ − f_r_^2^)/f_r_^2^ [16]_._ Figure 5e shows the underwater impedance data. The absence of any peaks or valleys might suggest weaker resonance behavior and much lower quality factors.

### 5.2. Vibration Measurements

**Figure 6:**
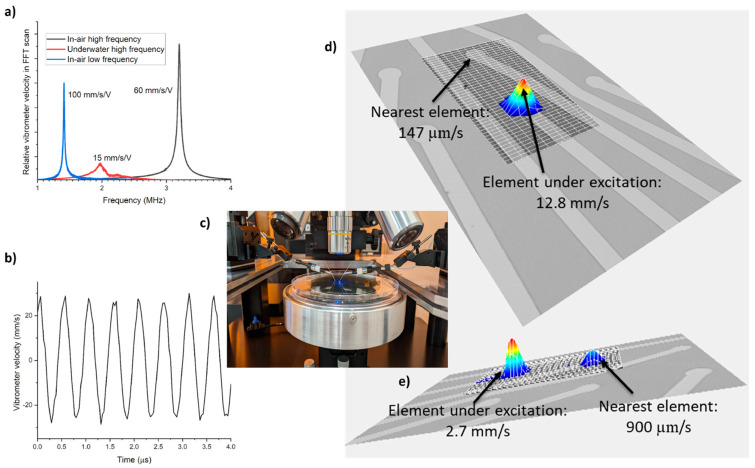
(**a**) Frequency response of a single PMUT with periodic chirp signal. (**b**) Underwater time domain response at 2 MHz with 2 V sinewave actuation. (**c**) Underwater measurement setup. Crosstalk (**d**) in air and (**e**) underwater.

The fabricated device was characterized in air and in DI water using a laser Doppler vibrometer (LDV). The in-air resonance frequency was recorded using periodic chirp excitation as 3.2 MHz (Figure 6a) with a transmission sensitivity of 60 mm/s/V. As demonstrated in the device design and simulations, the resonance frequency of a PMUT element can be controlled with the polyimide thickness. To experimentally demonstrate this, another array is fabricated with 3 µm thick polyimide, showing a resonance frequency of 1.4 MHz in the air with a higher sensitivity of 100 mm/s/V (Figure 6a). The measurements of eight PMUTs selected from this array show a small center frequency mismatch of 20 kHz (~1.5%), demonstrating good fabrication uniformity. Moreover, LDV studies can also be performed in liquid mediums, as demonstrated by several other studies on PMUTs [28,29]. Underwater, the resonance frequency and sensitivity dropped to 2 MHz due to medium damping (Figure 6a). The underwater sensitivity of the PMUT element is around 15 mm/s/V at 2 MHz, and this value was measured with sinewave excitation in a time-domain experiment (Figure 6b).

For crosstalk measurement, RMS velocities were recorded for the element under periodic chirp excitation and the adjacent unexcited element. A low in-air crosstalk (~1%) was recorded (Figure 6d). To the best of our knowledge, 6% in-air crosstalk is the lowest measured figure to date [28]. Meanwhile, underwater, coupled vibrations are observed in the nearby element (Figure 6e). The reason for this effect could be the closeness in the acoustic impedance of water (1.5 Mrayl) and polyimide (~3 Mrayl) [30]. However, it is hard to draw any final conclusions, as there is no available underwater crosstalk comparison in the literature on PMUTs. Crosstalk reduction can be further attempted with a few design changes such as implementing demonstrated island approaches [31]. These include the separation of individual PMUTs by etching the piezo layer in between and will be taken up in future work.

### 5.3. Underwater Acoustic Pressure Measurements

**Figure 7:**
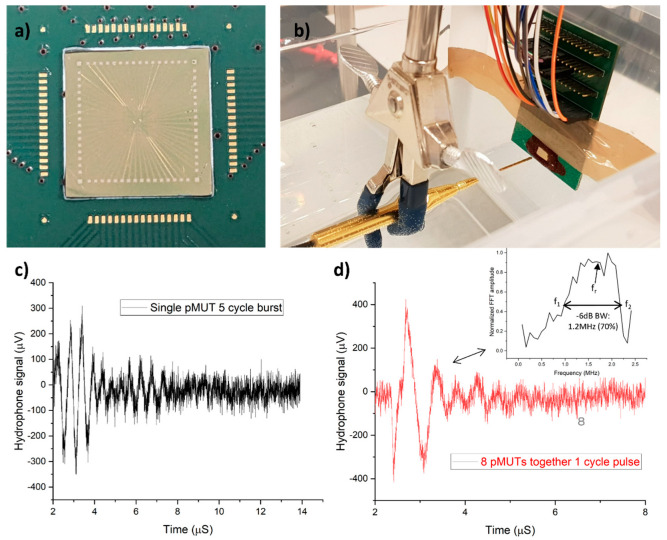
(**a**) PMUT array glued to the PCB before wire-bonding. (**b**) Underwater hydrophone setup with a PMUT array wire−bonded to a PCB. (**c**) The response of single PMUT to 5−cycle square wave burst signal at 5 mm. (**d**) Eight PMUT elements together with 1 cycle pulse at 1 cm.

The next measurements involved pulse characterization of the PMUT device underwater with a hydrophone (1 mm needle, Precision Acoustics). For this measurement, the PMUT array was glued to the PCB with epoxy (Figure 7a) and then wire bonded to the PCB. Bond pads along with the bond wires were sealed with epoxy. Figure 7b shows the setup for the hydrophone measurements. The hydrophone was aligned with the PMUT to obtain the maximum signal. However, while aligning, we ensured that the measurements were in the far field. The near field distance can be calculated as *a*^2^/λ and is about 3 µm, where *a* is the PMUT element radius and λ is the wavelength of the propagating ultrasound wave. Even if the array aperture is considered with a 1 mm radius, the distance is about 1 mm, ensuring the measurements are far field. The actuation amplitude was set as 20 V_p−p_. Square wave pulses at 2 MHz were applied with the HMF2525 function generator (Rohde & Schwarz) in this experiment. This signal generator can provide pulses up to 12.5 MHz with a rise time as small as 8 ns. Moreover, the hydrophone used has a flat response in the bandwidth of 15 MHz starting from 200 kHz. The case where the oscilloscope was used was similar. These suggest that the bandwidth of the acquired signal depends only on the drive signal and our PMUT transducer whereas the instrumentation is close to ideal. Hence, the true bandwidth can be calculated by deconvolving the acquired signal from the drive signal.

The first measurement concerned a single PMUT element. Figure 7c shows the response of a single PMUT to five cycles of a square wave burst signal at 2 MHz. It measures a transmission response of 400 Pa at 5 mm, i.e., 40 Pa/V. This suggests that focusing all 64 elements of the array together can produce approximately 20 kPa at the current impedance setting. However, with impedance matching, it is possible to generate even higher pressures. Still, the response can be considered to be on the lower side [14]. The lower response can be explained as follows: (1) It is the pressure generated by a single PMUT element and hence a very small vibrating aperture, resulting in a lower response. (2) It does not show much ringing, meaning a wide bandwidth and a wide bandwidth means that its vibration is damped significantly. Moreover, widening the bandwidth using a polyimide passive layer comes at the cost of a lower bending moment. Therefore, a lower vibration amplitude and, consequently, a lower output pressure are obtained. (3) Although 20 V_p−p_ is applied at the function generator, a smaller voltage appears across the PMUT due to an impedance mismatch and further reduces the pressure.

Note that the measured pressure of 40 Pa/V is in line with the simulated acoustic pressure (Figure 2h) and hence also with the simulated membrane displacement of 1.45 nm/V (Figure 2c). From the LDV measurement of 15 mm/s/V velocity, the membrane displacement can be calculated by division with angular frequency, and it comes out to be ~1.3 nm/V, which is close to the simulated membrane displacement at a similar resonance frequency, around 2 MHz. This shows that the LDV measurement is also in line with the hydrophone measurements. Moreover, the surface pressure (*p_s_*) of the PMUT element at 2 MHz can be theoretically calculated to be 9.2 kPa/V, which matches the simulation result of around 9.5 kPa/V in Figure 2b. The surface pressure, *p_s_*, of the PMUT is given by the equation below:

$$p_{s}=v.x.p.c$$

Here, *v* is the peak membrane velocity at the PMUT center, and the acoustic impedance is given by *x.ρ.c*. *ρ* and *c* are the density and acoustic velocity of water, respectively; *x* is related to the radiation resistance and reactance of a baffled circular piston and depends on *k.a*, where *k* is the wave number and *a* is the radius of the membrane [24]. *k.a* is 0.4 in our case and *x* is about 0.4 for a *k.a* of 0.4. *x* is related to the radiation resistance and impedance (can be calculated using complicated integrals, Bessel and Struve functions, the resulting graphs for which are visualized in [24].). The surface pressure compares well with state-of-the-art AlN PMUTs on SOI wafers (Table 3).

The second measurement was carried out by exciting eight PMUT elements together with one cycle pulse at 1 cm (Figure 7d). The distance was increased so that the combined effect of eight PMUTs translated into the measurement. This measurement shows a pressure of 500 Pa. Due to no focusing, output power limitations from the signal generator, and insufficient excitation energy from one cycle pulse, only 500 pa was obtained. The FFT of the time-domain response was divided by the FFT of one cycle square wave pulse for bandwidth calculation. The result after division is in the inset of Figure 7d and suggests that the transducer has a center frequency of around 1.7 MHz and a −6 dB bandwidth (BW) of 1.2 MHz, which is 70% of the center frequency, comparable to the most recent bulk-piezoelectric-based ultrasound imagers [35,36]. The fractional bandwidth was calculated as f_r_/(f_2_ − f_1_), where f_r_ is the center frequency of 1.6 MHz and f_2_ and f_1_ are −6 dB points at which the recorded FFT magnitude is half of the maximum value, as indicated in the inset of Figure 7d.

**Figure 8:**
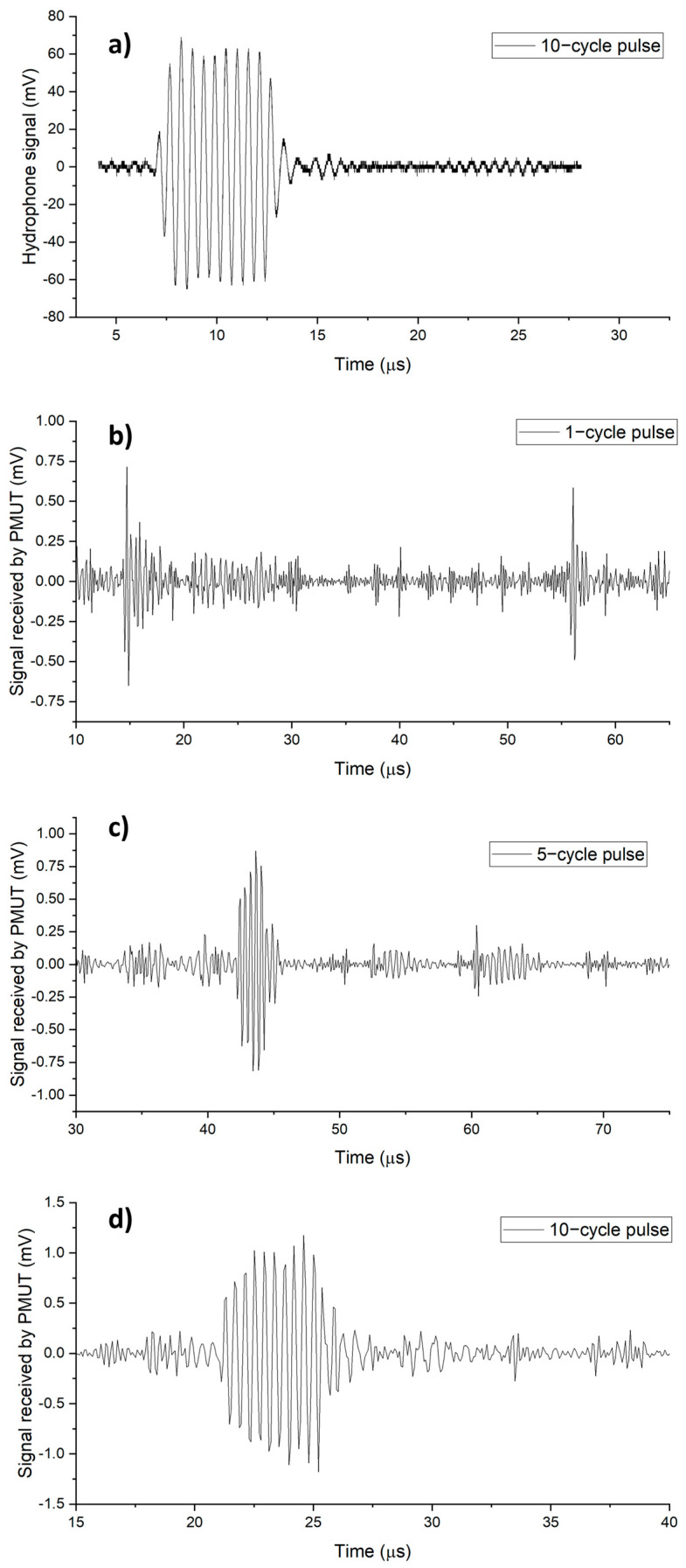
Characterization of PMUT receive response: (**a**) Signal measured via hydrophone. Signal measured via PMUT in response to (**b**) 1−cycle, (**c**) 5−cycle, and (**d**) 10−cycle pulses.

Lastly, to measure the receive sensitivity of the PMUT transducer, a single-element wide-band transducer (Precision Acoustics, UK) was used to transmit a burst signal at 2 MHz. The transmitted signal was measured by the calibrated hydrophone and the PMUT transducer. The measured hydrophone signal (Figure 8a) amounted to a 73 kPa pressure. Figure 8b–d show the received signal of eight PMUTs connected in parallel in response to 1, 5, and 10-cycle sinusoidal burst signals, respectively. Since the same pressure levels were applied to the PMUTs (by maintaining the same distance and applied burst signal level), the receive sensitivity was calculated by dividing the voltage signal in response to the 10-cycle burst by 73 kPa pressure, and a receive sensitivity of about 15 mV/MPa was obtained for the PMUTs. This receive sensitivity was found to be on the lower side because of the high parasitic capacitance of the PMUT interconnects. Note that the pulse response of the single-element transducer utilized in this experiment is not short and has some ringing. However, a very similar response was captured by the PMUT channel and hydrophone, again a confirmation of the wide bandwidth demonstrated by this technology.

Table 4 provides a summary of the presented results with a comparison of the theoretical calculations, simulation results, and experimental findings. Although our PMUT design is not optimized for a particular application, very low in-air crosstalk between our PMUT elements could be interesting for fingerprint sensing, as lower crosstalk enables higher SNR and lateral resolution [37]. However, for fingerprint sensing, the resonance frequencies of the PMUTs should be pushed to a higher value. Moreover, the hydrophone data suggests a very wide fractional bandwidth, which makes the PMUT attractive for medical imaging applications as well. For medical imaging applications using ultrasound transducer arrays, resonance frequencies of several MHz and a fractional bandwidth from 40 to 80% are used [38]. Sadeghpour et al. tabulated the specifications of state-of-the-art PMUT arrays designed for imaging, which show pressure levels of about a few kPa to perform imaging in several mm ranges [14]. To use our wide-bandwidth PMUT array for medical imaging, focused ultrasound, and impedance matching are required to improve the transmit response. Moreover, the transmit and receive sensitivity can be improved through mechanical and electrical design optimizations and will be considered in future work.

## 6. Conclusions

Low-cost SOI-free technology of a PZT-based PMUT is described in this paper for an 8 × 8 fully addressed array with its design and fabrication and detailed characterizations. The polyimide membrane passive layer enables high resonance frequencies. It is shown that the resonance frequency can be easily tuned by controlling the thickness of the polyimide. The PMUT membranes are realized using backside DRIE with an oxide etch stop. The fabricated PMUT with 6 µm thick polyimide membranes showed a 3.2 MHz in-air frequency with a 3 nm/V sensitivity, the lowest interelement crosstalk of 1% between the PMUT elements, and a high effective coupling coefficient of 14%. Additionally, a pressure response of 40 Pa/V at 5 mm was measured underwater using a hydrophone while exciting a single PMUT element. The single-pulse response captured using the hydrophone suggests a 70% −6 dB fractional bandwidth of the center frequency, 1.7 MHz. With some optimization, this technology can be applied in imaging and sensing applications in shallow-depth regions.

## Figures and Tables

**Table 1:** Crucial design parameters for the PMUT.

Parameter	Relevant for	Chosen Range
Membrane diameter	Resonance frequency, acoustic pressure	100 µm
Array size	Targeted application areas, for instance, affect imaging footprint in biomedical imaging	8 × 8 (2 mm × 2 mm)
Array pitch	Lateral resolution	300 µm
Resonance frequency	Chosen based on application, also relevant for axial resolution in imaging	2–2.5 MHz

**Table 2:** Details of the materials used for the PMUT technology.

Material	Function	Thickness (µm)	Young’s Modulus (GPa)	Density (Kg/m^3^)	Poisson’s Ratio	Deposition Method
Polyimide	Membrane passive layer	6	8.5	1420	0.34	Spin coating
PZT	Piezo layer	1	80	7500	0.3	Sol–gel
Pt	Top, bottom electrodes	0.2	168	21,400	0.39	RF sputtering
Silicon oxide	Etch stop for DRIE	0.5	70	2200	0.17	Thermal wet oxidation
Silicon	Membrane sidewall	525	140	2330	0.265	--

**Table 3:** Surface pressure comparison with the state-of-the-art PMUTs.

Reference	[32]	[33]	[34]	This Work
Technology	SOI, AlN	SOI, AlN	SOI, PZT	Conventional silicon wafer with polyimide membrane, PZT
Surface pressure (kPa/V)	4.9	2.9	27	9.2

**Table 4:** Summary and comparison of the presented results.

Parameter	Theoretical Calculation	Simulation Result	Experimental Findings
In−air resonance frequency	4.35 MHz		3.2 MHz
Underwater resonance frequency	2.34 MHz	1.8–2 MHz	1.7 MHz (70% fractional bandwidth
Underwater membrane displacement		1.45 nm/V	1.3 nm/V (3 nm/V in−air)
Underwater surface pressure	9.2 kPa/V (Calculated based on the measured membrane displacement underwater)	9.5 kPa/V	
Transmit pressure sensitivity at 5 mm		32 Pa/V	40 Pa/V (15 mV/MPa as a receiver)
In-air effective coupling coefficient and interelement crosstalk		14%, 1% respectively (Exp)	

## Data Availability

Not applicable.
